# Elevated fasting glucose levels associated with *H. pylori* acute gastritis: an observational study

**DOI:** 10.25122/jml-2025-0092

**Published:** 2025-09

**Authors:** Ioana Alexandra Cardos, Catalina Danila, Razvan Chirla, Ovidiu Laurean Pop, Andreea Camarasan, Simona Cavalu

**Affiliations:** 1 Doctoral School of Biomedical Sciences, Faculty of Medicine and Pharmacy, University of Oradea, Oradea, Romania; 2 Faculty of Medicine and Pharmacy, University of Oradea, Oradea, Romania

**Keywords:** *H. pylori*, glycosylated hemoglobin, fasting glucose level, gastritis, histopathology, MALT, Mucosa-associated lymphoid tissue, *H. pylori*, *Helicobacter pylori*, HbA1c, Glycated hemoglobin, RUT, Rapid urease test

## Abstract

*Helicobacter pylori* (*H. pylori*) is one of the world’s most prevalent infections, being responsible for 90% of gastric MALT lymphomas along with multiple other extra-gastric manifestations. Its role in insulin resistance and glycemic metabolism has been debated in the last few years. The study included a retrospective analysis of 131 patients with dyspeptic symptoms who underwent gastroscopy with biopsies in two hospitals in Northwestern Romania. Our study analyzed the overall prevalence of *H. pylori* infection, its association with high glycemic values and glycosylated hemoglobin values, as well as histopathology results and their association with modified glycemic values. Fasting glucose levels were higher in patients with *H. pylori* than in patients without *H. pylori* (OR = 3.3; 95% CI, 1.6–6.8; *P* = 0.001). High HbA1c levels were associated with *H. pylori* infection (OR = 4.1; 95% CI, 1.9–8.7; *P* < 0.001). Histologically confirmed acute gastritis due to *H. pylori* was associated with high fasting glucose levels (OR = 8.3; 95% CI, 1–68; *P* = 0.028), and more specifically with antral acute gastritis (OR = 16.4; 95% CI, 1–290; *P* = 0.007), while no association between confirmed chronic gastritis and high fasting glucose values was found. Within the limitations of this study, our results support the findings that *H. pylori* infection represents a risk factor for prediabetes, highlighting the need for special attention to be given to those vulnerable patients. To fully understand the involved mechanisms and the potential therapeutic strategies and management implications, further investigations are required.

## Introduction

*Helicobacter pylori* (*H. pylori*) is one of the most prevalent infections worldwide. In adults, its prevalence has declined from 52.6% (95% CI, 49.6%–55.6%) before 1990 to 43.9% (95% CI, 42.3%–45.5%) between 2015 and 2022. However, in children and adolescents, no significant decrease in prevalence has been observed [1].

This gram-negative, spiral-shaped pathogenic bacterium exclusively colonizes the stomach epithelium, resulting in peptic ulcer disease, chronic gastritis, or gastric cancer [2]. *H. pylori* infection is the etiological agent of 90% of gastric mucosa-associated lymphoid tissue (MALT) lymphomas. Furthermore, it has been demonstrated that the incidence of MALT lymphomas decreases with effective eradication therapy and that many affected individuals experience regression following treatment [3].

Although it primarily colonizes the gastric epithelium, *H. pylori* infection has been widely studied as a risk factor for various pathologies, including hematologic, cardiac, metabolic, neurological, and dermatological issues [4]. The associated inflammatory response depends on both host immune factors and bacterial virulence determinants. Notable pathogenicity features include the *cag* pathogenicity island, vacuolating cytotoxin A, and pathogen-associated molecular patterns such as lipopolysaccharides and flagellin, all of which promote inflammation [5]. Both innate and adaptive immunity are essential for the host's immunological responses because they activate receptors on immune cells, leading to the production and release of a variety of proinflammatory cytokines [6,7].

Recent studies have highlighted the role of *H. pylori* in dyslipidemia, insulin resistance, and type 2 diabetes mellitus (T2DM) [8,9].

Patients with persistently elevated glycemic levels eventually develop type 2 diabetes. Its complications are considered serious health burdens, with an escalating prevalence in recent years [10]. Insulin resistance, chronic inflammation, insufficient insulin secretion (resulting from compromised pancreatic beta cells), glucose toxicity, and lipotoxicity are pathogenic processes associated with diabetes mellitus [2]. *H. pylori* infection is more common in patients with T2DM than in healthy individuals [11]. Although the complete mechanism is unknown, several cytokines, including C-reactive protein, tumor necrosis factor, and interleukin-1β, are upregulated in *Helicobacter pylori* infections, leading to chronic, low-grade inflammation that may impact insulin action and pancreatic β-cell secretion. Additionally, *H. pylori*-induced gastritis may alter the secretion of gastric hormones, including somatostatin, leptin, ghrelin, and gastrin, thereby influencing glucose homeostasis and insulin sensitivity [12,13].

This retrospective observational study aimed to evaluate the correlation between *H. pylori* infection and higher fasting glucose and glycated hemoglobin (HbA1c) levels in patients with dyspeptic symptoms who underwent endoscopic examination in two tertiary health centers in Northwestern Romania between 2020 and 2024.

## Material and Methods

### General criteria

This retrospective analysis included 131 patients evaluated at the County Emergency Clinical Hospital in Oradea and the Regional Hospital of Salonta between 2020 and 2024. Inclusion criteria required participants to be over 18 years of age, present with dyspeptic symptoms, and undergo gastroscopy with biopsies and blood tests. Patients were either non-diabetic or diagnosed with type II diabetes; no patients with type I diabetes were included.

### Endoscopy

Endoscopic examinations were performed using an Olympus Exera II CV 165 endoscope and an Olympus Optera II endoscope, respectively, conducted by two experienced endoscopists. The endoscopic findings considered in this study were gastric polyps, intestinal metaplasia, atrophic gastritis, chronic and acute gastritis, gastro-duodenal ulcer illness, and gastric neoplasia.

### Helicobacter pylori diagnosis

The diagnosis of *H. pylori* infection was established using the rapid urease test (RUT; AMA Co Ltd., Lehmuskatu, Finland), which has a reported sensitivity of 90% and a specificity of 95–100%.

### Histopathology

The histological examination was the first method used to identify an *H. pylori* infection.

All biopsies were analyzed by two skilled pathologists using hematoxylin-eosin and Giemsa staining (Epredia-USA; Portsmouth, NH, USA). The specimens were assessed using the Houston-updated Sydney protocol [14].

### Laboratory exams

All blood samples were examined using the Abbott Alinity TM System apparatus (Abbott GmbH, Wiesbaden, Germany). All samples were taken from patients who were fasting, considering normal fasting glucose levels of less than 106 mg/dL and normal glycosylated hemoglobin levels of less than 5.7%.

### Statistics

Statistical analysis was performed using IBM SPSS Statistics for Windows, version 30.0.0.0 (IBM Corp., Armonk, NY, USA). The Fisher’s exact test and the Fisher–Freeman–Halton test were used to assess correlations between categorical variables, while the Mann–Whitney U test was applied to compare distributions of continuous variables. Odds ratios (ORs) and 95% confidence intervals (CIs) were calculated using the MedCalc online calculator.

## Results

### *H. pylori* prevalence and socio-demographic patient features

**Figure 1:**
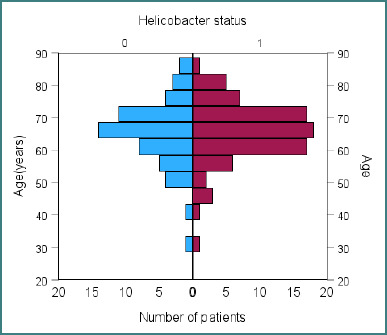
*H. pylori* prevalence according to age (0 = *H. pylori* negative, 1 = *H. pylori* positive patients). The mean age was 65.06 years for *H. pylori*-negative and 65.65 years for *H. pylori*-positive patients.

Among the 131 patients included, the overall prevalence of *H. pylori* infection was 59.53%. Of these, 58.02% were from urban areas and 41.98% from rural areas, with no significant correlation between residence and *H. pylori* prevalence. Likewise, *H. pylori* infection did not correlate with gender (*P* = 0.21) or age (Figure 1).

### Diabetes and *H. pylori* infection

Although the prevalence of diabetes in the dyspeptic cohort was high (53.43%), *H. pylori* infection was not significantly associated with T2DM (*P* = 0.285, Fisher’s exact test; OR = 1.52; 95% CI, 0.7–3.1).

### Laboratory tests correlations

#### Fasting glucose levels and *H. pylori* status

Fasting hyperglycemia was more prevalent in *H. pylori*–positive patients than in *H. pylori*–negative patients (*P* = 0.001; OR = 3.3; 95% CI, 1.6–6.8). This indicates that patients with fasting glucose levels greater than 106 mg/dL had 3.3 times higher odds of testing positive for *H. pylori*.

**Figure 2:**
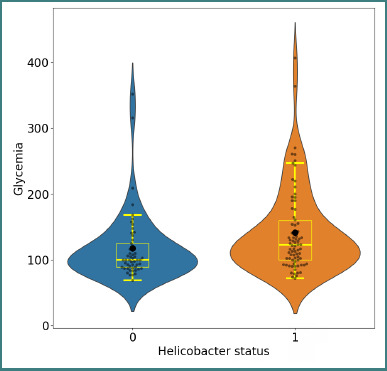
Distribution of fasting glucose values in *H. pylori*–negative and *H. pylori*–positive patients. The means (black dots), medians, and quartiles are indicated. Mann–Whitney U test, P = 0.004.

As shown in Figure 2 and Table 1, patients with *H. pylori–positive status* had higher mean and median fasting glucose values compared with those with *H. pylori–negative status*.

**Table 1:** Fasting glucose values in *H. pylori*–negative and positive patients

*H. pylori* status	Mean	*n*	Std. Deviation	Median
Negative	117.45	53	52.380	100.00
Positive	141.26	78	64.028	122.50
Total	131.63	131	60.525	110.00

#### Fasting glucose levels and *H. pylori* status according to gender

There was no statistically significant correlation between glycemia levels and gender, although men with *H. pylori* tended to have higher fasting glucose levels than women (as shown in Table 2) and were more likely to have fasting glucose levels higher than 106 mg/dl (OR = 1.4; 95% CI, 0.5–3.8). When analyzed by gender, men with *H. pylori* had higher hyperglycemia prevalence than women without *H. pylori* (*P* = 0.006), although in women, this correlation did not reach statistical significance (*P* = 0.09).

**Table 2:** Distribution of glycemic mean and median values according to *H. pylori* status and gender

Female patients' distribution of glycemia				
*H. pylori* status	Mean	*n*	Std. Deviation	Median
Negative	130.79	28	67.081	102.50
Positive	135.64	50	56.649	119.50
Total	133.90	78	60.213	114.00
**Male patients' distribution of glycemia**				
***H. pylori* status**	**Mean**	***n***	**Std. Deviation**	**Median**
Negative	102.52	25	21.038	100.00
Positive	151.29	28	75.531	124.00
Total	128.28	53	61.404	110.00

#### Glycosylated hemoglobin levels and *H. pylori* status

**Figure 3:**
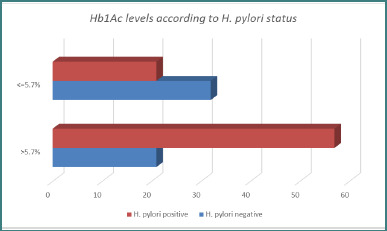
Proportion of patients with elevated HbA1c according to *H. pylori* status

Patients with *H. pylori* had significantly higher HbA1c levels compared with those who were *H. pylori–negative* (Figure 3). An HbA1c >5.7% was strongly associated with *H. pylori* infection (*P* < 0.001; OR = 4.1; 95% CI, 1.9–8.7), indicating 4.1-fold increased odds of testing positive for the bacterium.

**Figure 4:**
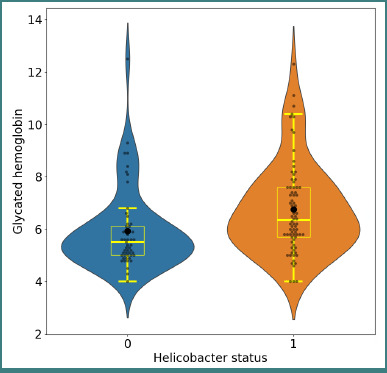
Distribution of HbA1c values in *H. pylori*–negative and positive patients. Mann–Whitney U test, *P* < 0.001

High HbA1c values were more common in positive *H. pylori* patients than in negative ones, as depicted in Figure 4.

#### HbA1c levels and *H. pylori* status according to gender

No overall correlation was observed between high HbA1c values and gender (*P* = 0.28). However, when analyzed separately, both female and male patients with *H. pylori* infection had significantly higher HbA1c compared with their *H. pylori*–negative counterparts (women: *P* = 0.022; men: *P* = 0.013). The mean and median HbA1c values are shown in Table 3.

**Table 3:** Distribution of HbA1c mean and median values according to *H. pylori* status and gender

Female patients' distribution of HbA1c				
*H. pylori* status	Mean	*n*	Std. Deviation	Median
Negative	6.250	28	1.8841	5.700
Positive	6.980	50	1.9210	6.300
Total	6.718	78	1.9280	6.000
**Male patients' distribution of HbA1c**				
***H. pylori* status**	**Mean**	***n***	**Std. Deviation**	**Median**
Negative	5.584	25	.8933	5.300
Positive	6.332	28	1.2576	6.350
Total	5.979	53	1.1540	5.600

### Histopathology results

#### Chronic gastritis and fasting glucose levels

Most biopsies showed mild to moderate inflammation according to the Houston-updated Sydney protocol. Chronic gastritis due to *H. pylori* was the most frequent histological finding, but showed no significant association with elevated fasting glucose, either in diabetic patients (*P* = 1.0) or in non-diabetic patients (*P* = 0.128)

#### Acute gastritis and fasting glucose levels

In contrast, histologically confirmed acute gastritis was significantly associated with high fasting glucose levels (OR = 8.3; 95% CI, 1–68; *P* = 0.028). However, this association was not statistically significant in patients with diabetes (OR = 5.0; 95% CI, 0.3–96; *P* = 0.31; Table 4).

**Table 4:** Prevalence of acute gastritis according to diabetic status and fasting glucose levels in *H. pylori*–positive patients

Diabetic status			High fasting glucose		Total
			Absent	Present	
Non-diabetic	Acute gastritis-histology	Absent	20.27%	12.16%	32.43%
		Present	1.35%	6.75%	8.1%
	Total		21.62%	18.91%	40.54%
Diabetic	Acute gastritis-histology	Absent	9.45%	37.83%	47.29%
		Present	0%	12.16%	12.16%
	Total		9.45%	50%	59.45%
Total	Acute gastritis-histology	Absent	29.72%	50%	79.72%
		Present	1.35%	18.91%	20.27%
	Total		31.08%	68.91%	100%

When stratified by location, antral acute gastritis was the main driver of this association (OR = 16.4; 95% CI, 1–290; *P* = 0.007). Non-diabetic patients with elevated fasting glucose had a significant association with antral acute gastritis (OR = 19.1; 95% CI, 1–384; *P* = 0.014), whereas diabetic patients did not (OR = 4.3; 95% CI, 0.2–84; *P* = 0.32).

## Discussion

Globally, *H. pylori* is a highly prevalent infection that is influenced by several variables, including age, sex, socioeconomic status, location, diet, and lifestyle [15]. According to certain studies, Romania has a comparatively high frequency of *H. pylori* infection, which aligns with patterns observed in many Eastern European countries. However, precise prevalence rates can differ depending on the population under study, the detection techniques used, and the geographical regions of Romania. In a 2024 study, the prevalence in southern Romania was 28.2% (95% CI, 24–32.6%), and the overall prevalence was 27.1% (95% CI, 23.9–30.6%) [16]. A 2020 study conducted in Northwestern Romania, based on serological testing, reported that 40% of dyspeptic patients were infected with or had previously been infected by *H. pylori*, suggesting a decreasing trend compared to earlier years [17].

Our study showed an overall prevalence of 59.53%. This difference may be explained by a combination of factors, including the fact that all included patients presented with dyspeptic symptoms, which are frequently associated with *H. pylori* infection. A 2021 study reported an even higher prevalence, with 66.6% of dyspeptic patients testing positive for *H. pylori* [18]. To ensure accurate diagnosis and appropriate treatment, the Rome criteria emphasize the importance of excluding *H. pylori* infection in patients with dyspeptic symptoms. Identifying and treating such underlying conditions can significantly improve patient outcomes [19].

One important aspect to consider is that all patients in our study were tested using the rapid urease test. With its speed, accuracy, and relatively low cost, RUT remains a valuable diagnostic tool for *Helicobacter pylori* infection. It plays a crucial role in guiding therapeutic decisions and enhancing outcomes in patients with gastric symptoms. Currently, RUT is recommended as a first-line diagnostic option when there is a clear indication for digestive endoscopy and no contraindications to obtaining biopsies [20,21].

Our study demonstrated an association between hyperglycemia and *H. pylori* infection in both male and female patients. Although the precise mechanisms linking *H. pylori* infection and metabolic disorders, such as hyperglycemia, are not yet fully understood, emerging evidence suggests a potential connection. A 2023 study conducted in China on 18,164 patients found that *H. pylori* infection was an independent risk factor for elevated blood glucose in non-diabetic individuals. Compared with eradicated infection (*P* = 0.007) and persistently negative subgroups (*P* = 0.029), mean glycemic values were significantly higher in the persistent infection group [22]. Given the potential associations between *H. pylori* infection and hyperglycemia, healthcare providers may consider screening for *H. pylori* in patients with unexplained hyperglycemia or metabolic syndrome.

Contrary to other studies conducted, we did not find an association between T2DM and *H. pylori* infection. Only a few studies support this observation. For example, a study published in 2001 in the *American Journal of Gastroenterology*, which included 429 patients, reported no link between *H. pylori*, diabetes, or upper gastrointestinal symptoms in diabetic patients [23]. Although numerous authors emphasize a potential association, there are currently no clinical recommendations suggesting screening for *H. pylori* in individuals with T2DM solely based on a possible relationship between the two conditions [24,25].

The connection between elevated HbA1c levels and *H. pylori* infection remains an area of active research. Our study identified a significant association between infection and higher HbA1c values, a finding consistent with several international studies [26-28]. Nevertheless, despite evidence suggesting that *H. pylori* infection may contribute to elevated HbA1c levels, routine screening for *H. pylori* is not currently recommended in patients with elevated HbA1c levels.

Acute gastritis is characterized histologically by inflammatory cell infiltration of the gastric mucosa, predominantly polymorphonuclear leukocytes within the lamina propria, which may extend into the glandular lumina or the submucosa, depending on the lesion severity. Whether blood glucose levels rise at the onset of infection has not yet been investigated. However, the diagnostic accuracy of histology is influenced by a number of parameters, including the location, size, and quantity of samples, staining techniques, proton pump inhibitors, antibiotics, and the experience of the examining pathologist [29].

A previous study investigated the association between HbA1c levels and endoscopic diagnosis, as well as the inflammatory response in *H. pylori* infection, concluding that this association is not related to the presence of *H. pylori* but rather depends on the extent of bacterial colonization and the degree of chronic gastritis [30]. However, this study did not analyze acute lesions and high blood sugar.

Despite the insights provided by our study, several limitations must be acknowledged. The cross-sectional design of the present study is a limitation, as it only allows us to establish associations without being able to define a cause-and-effect relationship. The number of enrolled patients was relatively small, as it was challenging to recruit patients meeting all the inclusion criteria. Hence, the study population may not be fully representative of the general population. Additionally, patient selection may be considered biased, as only symptomatic patients referred for gastroscopy were enrolled, rather than patients undergoing routine examinations. The diagnostic method for *H. pylori* is another limitation, as all patients were diagnosed using the RUT test.

While the exact relationship is still under investigation, *H. pylori* infection may be associated with increased insulin resistance, as reflected by a higher homeostatic model assessment (HOMA) index, which could contribute to metabolic issues [31,32]. Unlike other studies [33], we did not assess the HOMA index in our cohort, nor did we have access to detailed patient histories or risk profiles. Another limitation of our study is that medication use and treatment compliance were not considered, both of which could have influenced the observed outcomes.

Given these limitations, further research is needed to clarify the associations between *H. pylori* infection and hyperglycemia, to elucidate potential causal mechanisms, and to determine whether healthcare providers should consider screening for *H. pylori* in patients with unexplained hyperglycemia or metabolic syndrome.

## Conclusion

In our study, the overall prevalence of *H. pylori* infection was 59.53%, with no correlation observed between infection and gender, age, or environment. Fasting glucose levels were significantly higher in patients with *H. pylori infection* than in those without it. High HbA1c levels were associated with *H. pylori* infection regardless of gender. Histologically confirmed acute gastritis due to *H. pylori* was associated with high fasting glucose levels; antral acute gastritis was the main contributor to this correlation. No association was found between confirmed chronic gastritis and fasting glucose values. Within the limitations of this study, our results support the findings that *H. pylori* infection represents a risk factor for prediabetes, highlighting the need for special attention to be given to those vulnerable patients.

## Data Availability

All data are available in the archive (database) of the Clinical County Emergency Hospital of Oradea and Salonta Regional Hospital, Bihor County, Romania.
